# Delayed discharges from a tertiary teaching hospital in Israel- incidence, implications, and solutions

**DOI:** 10.1186/s13584-020-00425-x

**Published:** 2020-11-24

**Authors:** Gidon Berger, Danny Epstein, Meital Rozen, Avigdor Miskin, Michael Halberthal, Michal Mekel

**Affiliations:** 1 Department of Internal Medicine “B”, Rambam Health Care Campus, HaAliya HaShniya St. 8, 3109601 Haifa, Israel; 2 Hospital Management, Rambam Health Care Campus, Haifa, Israel; 3 The Ruth & Bruce Rappaport Faculty of Medicine, Technion-Israel Institute of Technology, Haifa, Israel; 4 Department of Internal Medicine “H”, Rambam Health Care Campus, Haifa, Israel; 5 Geriatric Medicine Service, Rambam Health Care Campus, Haifa, Israel

**Keywords:** Delayed discharge, Alternate level of care, Length of stay, Acute care

## Abstract

**Objectives:**

The Israeli health system is facing high workloads with average occupancy in certain hospital wards of around 100%. Since there is a shortage of hospitalization beds in institutions for continuous, long-term care, transferring patients from the general hospitals’ wards is often delayed. This situation has many significant ramifications, to the waiting patients themselves, to other patients who are waiting to be treated and to the entire organization. In this study, we describe the phenomenon of the “detained patients” - its extent, characteristics, significance, and possible solutions.

**Materials and methods:**

Rambam Health Care Campus is a tertiary medical center serving the population of the northern part of Israel. In recent years, the hospital management documents data regarding the “detained patients”. We reviewed hospital data of detained patients over a period of nine months. The data concerning adult patients awaiting transfer to an institution for continuous care, between May 2019 and January 2020, were obtained retrospectively from the computerized database of the social service.

**Results:**

During the study period, 12,723 adult patients were discharged. Of those, 857 patients (6.74%) were transferred to one of the facilities providing prolonged institutional care. For that group of patients, median inpatient waiting time from the decision to discharge until the transfer was 8 days (IQR 6–14), translating to 10,821 waiting days or 1202 hospitalization days per month. These hospitalization days account for 9.35% of the total hospitalization days during the study period. The “detained patients” were hospitalized in internal medicine wards (32%), orthopedic (30%), and neurology/neurosurgery (26%) departments. At any given moment, about 40 hospitalized patients were waiting for long-term care facilities.

**Conclusions:**

Health-care systems must adapt to the current patients’ case-mix to achieve optimal utilization of hospital beds and maximal operational efficiency. The number of long-term care beds should be increased, the coordination between general hospitals, health maintenance organizations and long-term facilities improved, and patients that may require long term care after the acute phase of their illness should be early identified and addressed. Meanwhile, establishment of organic units for waiting patients and reorganization of the hospital structure should be considered.

## Introduction

The Israeli health care system is characterized by a low number of hospital beds per 100,000 population and a high (close to 100%) acute care beds occupancy rate [1]. This problem is even more severe in tertiary hospitals and certain medical departments such as internal medicine, orthopedic surgery, general surgery, and neurology wards [2, 3].

Among the factors that determine the system load is the turnover of patients. This indicator is directly translated into the length of hospital stay and the occupancy of inpatient beds. Israeli hospitals have the second-highest occupancy rate in the Organization for Economic Co-operation and Development (OECD), after Ireland, and a very short average length of stay, in general, acute care hospitals [4]. The overloads lead to long waiting times in the emergency departments, impairment of the quality and safety of care, inefficient resource utilization, as well as staff burnout [5, 6].

A careful review of the reasons for such high occupancy indexes of acute care beds reveals that a significant portion of those beds is occupied by patients that do not require active acute in-hospital care. In fact, in recent years, a new type of hospitalized patient has formed in Israeli hospitals - the “detained patients”. These patients are patients that are admitted to the hospital due to an acute illness, but after overcoming the acute stage of their disease and attaining clinical stability they require either inpatient rehabilitation or long term care in nursing homes or facilities capable of caring for chronically ventilated patients. These patients stay in acute care hospitals while waiting for a place in a long-term continuous care institution.

In this article, we present the phenomenon of “the detained patients”- its extent, characteristics, implications, and possible solutions.

## Materials and methods

Rambam Health Care Campus in Haifa is a 1000-beds tertiary academic hospital serving over 2.4 million residents in northern Israel. According to hospital records, there are 80,000–90,000 inpatient admissions every year.

The data regarding hospitalized patients waiting for rehabilitation or prolonged continuous care facility between May 2019 to January 2020 were obtained from the computerized database of the hospital’s social service. Only adult patients (> 18 years old) were included in the study. The data were retrieved anonymously and included age, gender, health maintenance organization (HMO) affiliation, hospitalization ward, type of prolonged care facility the patient was waiting for (rehabilitation, complex nursing home, a facility for palliative and hospice care, sub-acute nursing home, and institution for chronically ventilated patients) and length of wait from the decision to discharge until transfer. Additional data included documentation of the need for hemodialysis and multidrug-resistant (MDR) bacteria carriers, as there is a limited number of facilities taking care of these patients. Hospitalization in these mentioned continuous care institutions is, by law, funded by governmental health insurance funds.

Patients’ characteristics were summarized with descriptive statistics. Qualitative variables were expressed as numbers (percentage). Mean (±standard deviation) and median (interquartile range, IQR) were used for the description of normally and non-normally distributed quantitative variables, respectively. Distribution normality was determined using histograms. Normally distributed values were compared using paired Student’s t-test while the Wilcoxon signed-rank test was utilized for non-normally distributed covariates. The Chi-squared test was used to analyze the differences between categorical variables. Two-tailed *p*-value< 0.05 was considered statistically significant. Data analysis was conducted with the Statistical Package for the Social Sciences, version 23.0 (IBM SPSS Statistics for Windows, Version 23.0. Armonk, NY: IBM Corp) and Microsoft Excel version 14.0 (Microsoft Corporation, Redmond, Washington).

The requirement for institutional review board approval was waived by the Institutional Review Committee of Rambam Health Care Campus.

## Results

During the study period, 12,723 adult patients were hospitalized at Rambam Health Care Campus for 3 or more days. These hospitalizations accounted for a total of 115,707 hospitalization days. Of these patients, 857 patients (6.74%) were awaiting transfer to another institution for continuous care for 1 day or longer before discharge. Those institutions included rehabilitation, complex nursing home, palliative and hospice care, sub-acute nursing home, or institutions for chronically ventilated patients.

The clinical characteristics of the patients included in the study are presented in Table 1.

**Table 1:** Clinical characteristics of patients included in the study

	Long care facility the patients are waiting for					
	Rehabilitation	Complex nursing care	Chronic mechanical ventilation	Sub-acute hospitalization	Hospice	Total
**Number of patients (%)**	668 (78%)	87 (10%)	45 (5%)	3 (0.4%)	54 (6%)	857
**Median age, years (IQR)**	70 (58–80)	71 (62–77)	68 (60–78)	67 (53–70)	71 (63–83)	70 (59–79)
**Male gender, n (%)**	361 (54%)	47 (54%)	36 (80%)	3 (100%)	22 (41%)	469 (55%)
**Carriers of MDR bacteria**	36 (5%)	21 (24.14%)	14 (31%)	0	4 (7%)	75 (9%)
**Dialysis patients, n (%)**	5 (0.8%)	4 (5%)	1 (2%)	0	0	10 (1%)

*MDR* Multiple drug resistant; *IQR* Interquartile range

Median waiting time was 8 days (IQR 6–14, range 4 to 267 days). The maximal waiting times were 267 days, 125 days, and 104 days. All three patients were MDR bacteria carriers. The cumulative number of waiting days was 10,821 hospitalization days during the 9 months period or 1202 hospitalization days per month. These waiting days represent 9.35% of the total hospitalization days during the study period.

The patients waited in the internal medicine wards (32%), orthopedic wards (30%), and in neurology and neurosurgery departments (26%). Sixty-nine percent were ‘Clalit’ health insurance fund policyholders and 21% were insured by ‘Maccabi’ HMO.

Median (IQR) waiting times were 18 (8–27) days for chronically ventilated patients; 15 (8–27) days for complex nursing facilities; 11.5 [7–19] days for palliative care ad hospice; 11 (10–31) days for sub-acute hospitalization and 8 (8–27) days for patients awaiting for rehabilitation. Interestingly, the waiting times for members of the ‘Clalit’ were significantly longer compared with patients from the other three HMO’s (9 vs. 7–8 days, respectively, *p* < 0.039).

Seventy-five patients carried MDR bacteria (9%) and their median waiting time was 2.5 times longer (21 vs. 8 days, *p* < 0.001). Patients on chronic hemodialysis treatment also waited significantly longer than patients not requiring such treatment (12 vs. 8 days, respectively, *p* = 0.045).

One hundred and one of 668 (15%) patients who waited for rehabilitation were eventually discharged home and a total of 163 patients (24.4%) were discharged to a different facility from that originally intended. This group included patients whose status changed during the waiting period from institutional rehabilitation to home rehabilitation or patients who preferred to be discharged home rather than continue and wait for the rehabilitation facility.

## Discussion

The shortage of facilities for continued care is a known problem in the Israeli health care system. In the current study we found that at any given moment, there were about 40 patients in Rambam Health Care campus waiting for a slot in one of the institutions for continued care, meaning that the detained patients occupy 4% of the hospital beds or close to a fifth of the beds in the internal-medicine division. As of today, we can say that at any given time in orthopedics and neurology/neurosurgery departments almost 20% of the beds are occupied by patients who are waiting for a bed in sub-acute care or long-term facility, mainly for rehabilitation. This number may be even higher as some additional patients are waiting for an available slot in self-funded institutions, like nursing homes and institutions for patients with cognitive impairment. Although the dimensions of this phenomenon may vary among different hospitals in Israel, depending on the availability of continuous care facilities in any specific geographic region, there is no doubt that a significant portion of acute care beds in tertiary hospitals is dedicated to the “detained patients”. Rambam Health Care campus charges HMOs $859,893 to $1.05 million per month, for patients experiencing a delay in discharge.

The aging of the Israeli population alongside with the concomitant rise in age-related diseases and disability leads to an increase in the number of patients requiring a bed in long-term facilities following hospitalization in a general hospital [4]. Delays in discharge from acute care hospitals constitute a challenge for many health care systems in the developed world. These delays have a substantial negative effect on emergency department crowding, availability of hospital beds for new admissions, quality of care, hospital costs, and staff burnout [2, 7, 8]. Patients who experience a delay in transfer to an appropriate long-term facility have an adverse clinical course, including an accelerated functional decline, depression, anxiety, social isolation, loss of independence and increased risk for hospital-acquired infections, unnecessary procedures [7–10].

Moreover, in the current operative mode of inpatient wards, the detained patients are given the same level of care as the other patients, although they do not have an active medical problem. Therefore, a problem of inefficiency arises due to the mismatch between resource allocation and the needs of this patient population, mainly shifting of medical staff (i.e., physicians) from ‘active’ patients to waiting patients. On the other hand, the waiting patients are also affected as their specific needs (i.e., nursing, physiotherapy, and social worker care) are ‘diluted’ with the different needs of the patients around them and therefore are not being met. And finally, the waiting patients restrict the hospital from fulfilling its goal as an ‘acute care’ institution.

The data regarding the described phenomenon is scant. Studies performed in Canada show that only 33 to 50% of days in the general hospital are for acute care [11, 12]. Another study, also performed in Canada, shows that in 30 day of hospitalization, 35% of the patients have no acute medical condition that requires acute medical care. Fifty-six percent of them are on the waiting list for a long-term care facility [8]. Irish health care system is facing the same difficulties [13]. The waiting times described by others are in concordance with our findings; in Canada and Ireland, the mean waiting time for the nursing home is almost 40 days [7, 13]. In a study conducted in a trauma center in the USA, one in 25 patents had on average 6 days of delay in discharge, mainly attributable to difficulties with patient placement, including an absence of rehabilitation or subacute hospital beds. Total hospital charges for excess days in the hospital for patients experiencing a delay in discharge were $2,455,703 per year [14].

In the United Kingdom (UK), where, similar to Israel, health care policy and budgets are set centrally by the government and administered locally by National Health Service organizations with delegated powers, data regarding delayed discharges is collected monthly at the national level [15, 16]. It is estimated that in 2016/2017 over 2.3 million bed days were lost due to delayed discharge and the number is increasing every year [17, 18]. The annual cost of this phenomenon has been estimated at $1.08 billion [17]. It has been reported that roughly a quarter of hospitalized patients in the UK do not meet acute care criteria. Both in-hospital reasons (such as awaiting consultant decision, procedure/investigation/results not meeting criteria for acute care) and out-of-hospital reasons (such as waiting for long term care allocation) were responsible for the delays [9]. The German health-care system is characterized by statutory health insurance and autonomous regional or employment-based sickness funds that provide insurance coverage for a great majority of the population [19]. Studies performed in Germany demonstrate that 28–33% of consecutive hospitalization days are inappropriate [20]. Most probably delayed discharges are responsible for a significant portion of these days.

A systematic review of studies dealing with delayed discharge problem in OECD countries found that the average cost of extra hospitalization days is $262–$741 per patient per day, translating into a cost of more than $131,000 per year for a regular ward of 30 beds. As much as 11 to 31% of total hospital costs could be saved by avoiding discharge delays [10].

The delayed discharges and prolonged waiting lists reflect a significant mismatch between the patients’ needs and the availability of the appropriate health care services, in this case, the low number of long-term care (LTC) beds. In 2017, the mean number of LTC beds in OECD countries was 47 beds per 1000 people 65 years old or older. In Israel, at the same time, there were only 23 such beds per 1000 elderly citizens. Moreover, this number dropped by 7.8 beds between 2007 to 2017 [21].

In 2019 there were 4046 licensed LTC beds in Israel- 772 beds dedicated to patients who need prolonged mechanical ventilation, 1735 complex nursing beds, 297 sub-acute beds, and 1242 rehabilitation beds dedicated to the elderly [22]. The current billing strategy for LTC facilities as well as the accounting rules between the HMO and general hospitals creates a financial conflict between the payer and the provider in light of the discrepancy between the cost of the hospitalization day and the actual expenditure on the service for the detained patients. These factors do not encourage the establishment of new LTC beds and do not motivate both sides to rapidly transfer patients waiting in general hospitals.

The shortage of LTC beds must be addressed by the Ministry of Health. In the long run, a definitive solution would be a significant increase in the number of LTC beds and improvement in the community services supporting patients discharged from hospitals with ongoing non-acute care needs. Other means for facilitating the discharge process include improving the discharge planning. The main components of effective discharge planning include early assessment of patient’s physiological, psychological, social, and cultural needs, early identification of patients that may require rehabilitation or prolonged care after the acute phase of their illness, development of personalized discharge strategy, tailored to the patient, caregiver, and community provider, and efficient implementation of the plan [23]. These necessitate improving the communication and coordination with HMOs and long-term facilities and increasing the number of trained social workers available in the general hospitals. Better and early understanding of the patient’s sociocultural perspective and family support structure may assist the staff to anticipate barriers to discharge. Investment in co-operation between health and social services in the UK (Community Care Act) significantly reduced the number of delayed transfers, mainly by reduction of social service delays [24]. In the United States, discharge planning is a legally mandated function for hospitals [23]. Discharge plans tailored to the individual patient were shown not only to reduce the length of hospital stay but also to reduce the readmission rates and increase patients’ and healthcare professionals’ satisfaction [25].

However, we suggest a complementary strategy. As occupying acute care beds is not the best option for patients and the healthcare system, we suggest establishing specially designed hospital units for the patients waiting for long-term care. Supposedly, it requires additional costs unavailable in the deficient health care system. However, we argue that the opposite is true as an establishment of conventional acute care units is much more expensive and stuff demanding. For example, from looking at LOS and turnover data, we can estimate that a new internal medicine department with 36 new beds will increase the total number of daily hospitalizations only by an average of 6–8 patients, assuming that some hospitalizations are the result of supply-induced demand, even less. Therefore, the addition of these beds will lead to only minor increase in patients’ turnover and will require a large financial investment. However, establishing a dedicated unit for the waiting patients will achieve the same effect in terms of turnover, provide a solution to all mentioned above problems created by patients waiting within the acute care wards, and will significantly cut the costs over the alternative. These units do not require a department director, a deputy director, 4 senior doctors, and 10 residents, but only limited medical and nursing staff, as these patients do not have acute active medical problems. On the other hand, these units should be staffed by a relatively large number of physiotherapists, nutritionists, and social workers. These units will reduce the exposure of patients to nosocomial infections and other hazards of a prolonged stay in the acute care department, enable the patients to be surrounded by their families most of the day in a much more relaxed environment, and allow a smoother transition to the long care facility. Such units will prioritize patients’ care rather than the organization, making the terms “detained patients” or “bed-blockers” a misnomer.

Community-based programs should be developed, as well. These programs may enable discharge for ambulatory care some of the patients waiting for a nursing home, palliative care, and rehabilitation with the support of transitional programs and increased community care.

Our study has several limitations. First, we did not collect information on institutional factors such as practice protocols that may also contribute to prolonged inappropriate patient stays. Second, the applicability of our findings to other healthcare environments and hospitals may be limited due to different system organization and funding planes.

## Conclusion

This study shows that patients waiting for long-term facilities in general hospitals contribute to high occupancy rates of acute care beds. The major reason preventing their discharge is the paucity of available and appropriate long-term resources of care. This complicated problem deserves a systemic, comprehensive, and complete solution. Future research should focus on developing strategies to better address the discrepancies between patients’ needs and available healthcare resources. The addition of specialized units that will treat the waiting patients, mainly in large hospitals, may be a part of the comprehensive solution.

## Data Availability

The datasets used and/or analysed during the current study are available from the corresponding author on reasonable request.

## Abbreviations

OECD: Organization for economic co-operation and development
HMO: Health maintenance Organization
MDR: Multidrug-resistant
IQR: Interquartile range
LTC: Long-term care
